# Structural and Functional Outcomes of Surgery for Lamellar Macular Holes with or without Epimacular Proliferations

**DOI:** 10.18502/jovr.v17i1.10169

**Published:** 2022-01-21

**Authors:** Ramesh Venkatesh, Arpitha Pereira, Kushagra Jain, Naresh Kumar Yadav

**Affiliations:** ^1^Department of Retina-Vitreous, Narayana Nethralaya Eye Hospital, Rajaji Nagar, Benguluru, India

**Keywords:** Epimacular Membrane Proliferation, Full-Thickness Macular Hole, Lamellar Hole Epithelial Proliferation, Lamellar Macular Hole, Surgery

## Abstract

**Purpose:**

To compare the clinical, optical coherence tomography (OCT) features, and surgical outcomes of lamellar macular hole (LMH) depending on the presence of epimacular membrane proliferation (EMPF).

**Methods:**

This retrospective chart review included 112 eyes with LMH. The patients were divided into two groups depending on the presence of EMPF. Group 1 had LMH without EMPF and Group 2 had LMH with EMPF. The best-corrected visual acuity was recorded and OCT scans were obtained.

**Results:**

Lamellar macular hole without and with EMPF was noted in 62 (55%) and 50 (45%) eyes, respectively. The presence of EMPF was associated with lower presenting visual acuity (*P* = 0.049), wider LMH size at the largest diameter on the horizontal scan (*P* = 0.001), thinner residual retinal tissue (*P* = < 0.0001), and larger IS-OS defects (*P* = < 0.0001) as compared to the non-EMPF group. Of the 112 eyes, 18 eyes underwent surgery for LMH. Seven eyes had EMPF and the remaining eleven did not have EMPF. The average follow-up time for patients post-surgery and under observation was 16.8 and 24.1 weeks, respectively. A significant improvement in visual acuity was noted in the operated eyes with no EMPF as compared to the eyes with EMPF (*P* = 0.008). Worsening visual acuity (*P* = 0.021) was noted in eyes with LMH associated with EMPF which did not undergo surgery. Eyes with LMH and no EMPF, which were not operated on showed a minimal negative change in visual acuity.

**Conclusion:**

LMH with EMPF showed a higher association with accompanying ellipsoid zone disruption. Better anatomical and functional outcomes were achieved in those eyes that underwent surgery for LMH with no presence of EMPF and ellipsoid zone defect.

##  INTRODUCTION 

Epimacular membrane proliferation (EMPF) was previously described as a “thick” epiretinal membrane (ERM) by Witkin et al in 2006, “dense non-tractional membrane” by Parolini et al in 2011, and then more commonly as the lamellar hole epithelial proliferation (LHEP) by Pang and associates in 2014.^[1,2,3]^ This different type of ERM seen in cases of lamellar macular holes (LMH) on high-resolution optical coherence tomography (OCT) was identified as a homogenous mass of medium reflectivity lying over the retinal surface.^[3]^ In 2015, Schumann et al renamed this phenomenon as “atypical epiretinal tissue”, because it occurred in conditions other than LMH, such as in a full-thickness macular hole (FTMH) condition.^[4]^ The commonly accepted hypothesis for the formation of LMH is that it arises from the ERM contraction, which then results in a tear in the inner retinal layers.^[2,5]^ During clinical examination, EMPF is identified as a yellow elastic jelly lying over the epiretinal surface and commonly associated with a thick non-contractile ERM. Its yellowish color is likely due to the xanthophyll pigment identified in histological analysis.^[2,6]^ Despite the absence of a clear mechanism for EMPF formation, the most likely theory is that it results from the migration of the retinal Muller glial cell.^[3]^ The association of the EMPF with higher rates of ellipsoid disruption and positivity to pan-keratin created another theory of the retinal pigment epithelial origin of the disease.^[4,7,8,9,10]^ It has been reported that patients with EMPF tend to have lower baseline visual acuities, greater external MH diameters, thinner residual retinal tissue, and higher rates of inner segment-outer segment (IS-OS) band disruption when compared to non-EMPF eyes.^[4,7,9]^ Visual acuity in eyes with LMH varies from having baseline normal vision to having lower visual acuities depending upon the integrity of the ellipsoid zone. A wide variety of anatomic and visual outcomes has been reported after the performance of vitrectomy for macular holes with and without EMPF presence. Marques et al reported no differences in visual performance or closure rates between the EMPF group and non-EMPF group after surgery or in the subset of patients who did not undergo treatment.^[11]^ One paper reported a significantly poor visual outcome for patients with LMH and EMPF after surgery,^[6]^ while another study showed an increase in area of EMPF and a decline in the visual function in eyes who were managed conservatively.^[12]^ The majority of retinal specialists in the Indian subcontinent tend to manage the cases of LMH conservatively either because of underlying ellipsoid layer integrity, better presenting visual acuity, or associated poor visual prognosis. Given the variable characteristics in the physical and structural properties of EMPF, there are no clear guidelines currently available regarding patient selection and timing of surgery in such eyes with LMHs. In addition, to the best of our knowledge, there is no reported literature from the Indian subcontinent describing this clinical entity in these eyes or discussing the treatment outcomes either from surgery or through conservative management. In this report, we intend to analyze the morphological changes and visual outcomes of the LMH cases that presented with EMPF, and describe the surgical outcomes. By comparing the clinical and surgical data from the LMH cases with and without EMPF, we intend to appreciate and understand better the significance of this unique epimacular proliferation. The objective of this study is also to make the readers aware of this clinical entity in clinical/OCT examinations and to encourage them to understand the relevance and impact it can have on the final outcome of the disease.

##  METHODS 

This study was approved by Narayana Nethralaya institutional review board and ethics committee (C-2019/01/003). This retrospective study was conducted at the retina clinic of a tertiary eye hospital in Southern India. In this study, a single observer (RV) reviewed the SD-OCT images acquired by the Spectralis, Heidelberg machine, which were saved in the folders labelled as lamellar macular hole and epiretinal membrane between the January 2011 and December 2018. The diagnosis of LMH was based on the updated criteria proposed by the International Vitreomacular Traction Study Group.^[13]^ According to the group, LMH is a non-full-thickness retinal defect seen at the macula. This defect is characterized by the presence of the following features on the SD-OCT: (1) an irregular foveal contour; (2) inner foveal defect; (3) intraretinal splitting, typically between the outer plexiform and outer nuclear layers; and (4) presence of a photoreceptor layer at the base of the hole. The OCT images were viewed to identify the presence or absence of EMPF in these eyes. EMPF was identified on the OCT imaging as a homogenous material of medium reflectivity arising from the outer retinal layers, crawling along the walls of the macular hole and lying on the epiretinal surface. The eyes were categorized into two groups for further analysis: (1) LMH with no EMPF and (2) LMH with EMPF. Demographic data records included age, gender, laterality, and Snellen visual acuity (VA) at presentation. Features on OCT which were recorded included presence of LMH, EMPF, IS-OS defect, and ERM. The macular hole size in LMH was measured as the widest horizontal diameter at the level of the middle retinal layers at the foveal center. The length of the IS-OS defect and thickness of residual retinal tissue in LMH were manually measured at the fovea using the calipers provided with the software. The LMHs observed on the OCT were further divided into tractional or degenerative types based on the classification proposed by Govetto et al.^[14]^ The tractional type was characterized by the schitic separation of neurosensory retina between outer plexiform and outer nuclear layers with an intact ellipsoid layer and was associated with tractional epiretinal membranes and/or vitreomacular traction at the fovea. The degenerative type was characterized by the presence of intraretinal cavitation, non-tractional epiretinal proliferation, a retinal "bump" and with an early ellipsoidal zone defect. In addition, other documented data included treatment and outcome of surgery for the LMH, postoperative VA, and anatomic status of the macular hole. The indications to operate in the LMH group were visual acuity < 6/12, patient who complained of metamorphopsia and had a presence of an epiretinal membrane. In the present study, we looked at patients with a minimum follow-up of eight weeks following surgery to analyze the outcomes. Successful macular hole closure was defined as the collapse of the excavation between the outer nuclear and outer plexiform layers while achieving a normal foveal contour.

### Surgical Technique 

All the surgeries were performed by a single surgeon (NKY). A three-port 23- or 25-gauge pars plana vitrectomy was performed. After core vitrectomy, intravitreal triamcinolone acetonide was injected to stain the posterior cortical vitreous and the posterior vitreous detachment was then induced. In cases where epimacular membrane was present, removal was performed. Care was taken to not forcibly peel the ERM from the edge of the LMH. A vitrectomy cutter was used to trim and leave the adherent epiretinal tissue at the margin of the hole. An attempt was made to remove the EMPF that was lying over the internal limiting membrane (ILM). A 0.1–0.2 cc of Brilliant Blue Green (BBG) dye was injected to stain the ILM to facilitate its removal from the macular area. If the ERM was difficult to identify, BBG-assisted ILM peeling was done from outside the ERM-covered area that was not stained. The ERM was then removed along with the ILM. Again, care was taken to not forcibly peel the ERM/ILM from the edge of the hole. Finally, air–fluid exchange was done and 15 % perfluoropropane (C3F8) or 20% Sulphur hexafluoride (SF6) gas was used for an endotamponade procedure. A minimum of seven days of prone positioning was recommended.

### Statistical Analysis 

Normal distribution of quantitative variables was checked using the Kolmogorov–Smirnov test. Snellen's vision data were converted to logarithm of minimum angle of resolution (logMAR) vision for statistical analysis. Categorical variables were labelled as numerical for easy analysis as in identification of the IS-OS defects, the ERM, and the hole closures. Value 1 indicated presence and value 0 absence of these findings. Categorical variables between the two groups were compared using the Chi-square test. The Mann–Whitney U-test was used to compare quantitative data between the two groups. Correlations between the presence of EMPF and other variables were determined using the Spearman correlation test. A correlation (*r*) value of 0 means no correlation between the two variables while values closer to –1 indicate strong negative correlation and values closer to +1 indicate strong positive correlation. For the analysis of surgical outcomes, the eyes were divided into two groups: (1) eyes with EMPF and (2) eyes without EMPF. Wilcoxon signed-rank test was applied for the comparison of VA changes in the two groups. All data were analyzed with GraphPad Prism software (version 8.1.1). *P*-values < 0.05 were considered statistically significant.

##  RESULTS

During the study period, a total of 112 eyes with LMHs were included. The number of eyes included in each group were: (1) Group 1 – Eyes with LMH and no EMPF (62, 55%); (2) Group 2 – Eyes with LMH and EMPF (50, 45%).

**Figure 1:**
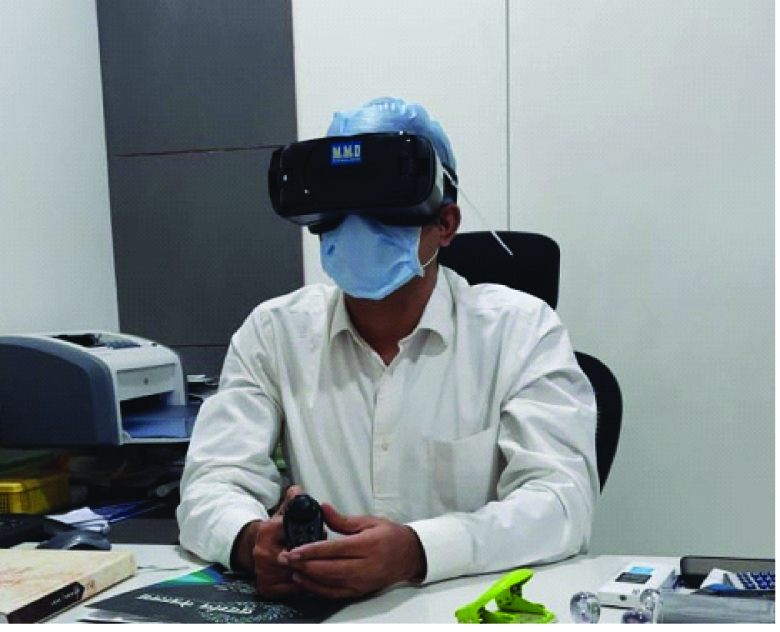
Lamellar macular hole (LMH) with EMPF. (a) Optical coherence tomography (OCT) image of a patient with LMH in right eye showing the presence of epiretinal proliferative tissue at the margin of the hole (white arrow) with presence of ellipsoid zone defect (red star). (b) Another OCT scan passing through a different section acquired on the same day demonstrating the extension of the proliferative tissue (yellow arrow) from the ellipsoid zone defect (red star) and then crawling along the walls of the macular hole to lie over the retinal surface.

The comparison of clinical and OCT findings of patients with lamellar macular hole presenting with and without EMPF is described in Table 1 and depicted in Figure 1.

Tractional types of LMH were identified in 25 (22%) eyes, the degenerative types of LMH in 81 (72%) eyes, and mixed variety in 6 (6%) eyes. EMPF was most commonly seen with the degenerative type of LMH (45/50, 90%) followed by the mixed type (5/50, 10%). ERM was absent in eight eyes with EPMF and LMH. Analysis of presence of EMPF with different OCT features showed strong positive correlations with the presence (*r* = 0.742) and size (*r* = 0.743) of IS-OS defects while strong negative correlation was noted with thickness of the residual retinal tissue (*r* = –0.641) present within the MH [Table 2].

Moreover, 18 of the 112 (16%) cases with LMH were treated surgically using the pars plana vitrectomy procedure. The remainder of the cases were managed conservatively. Of the 18 eyes which underwent surgery, 7 eyes had EMPF. Table 3 compares the clinical and OCT features of eyes with and without EMPF that were operated on. The width of the LMH (*p* = 0.027), size of the IS-OS defect (*p* = 0.002), and residual retinal tissue thickness (*p* = 0.000) showed statistically significant correlation between the two groups. The average follow-up period for patients post-surgery was 16.8 weeks. Single surgery hole closure was achieved in 4 (57%) eyes and 11 (100%) eyes in cases with and without EMPF, respectively. Of the remaining three eyes where surgical success was not achieved due to the development of a full-thickness macular hole, repeat surgery introducing a fluid-air exchange and silicone oil/intraocular gas tamponade was performed in all the eyes. In two eyes the hole closed while in one eye the hole remained open despite the second surgery. In the observation group, 43 of the 94 (46%) eyes, which were managed by observation, showed EMPF on the SD-OCT scans. However, by the end of the final follow-up visit, an additional 16 eyes showed development of EMPF, thus increasing the number to 59 (63%) eyes for those who were managed conservatively. The average follow-up period for patients under observation was 24.1 weeks.

The mean preoperative visual acuity in eyes with EMPF and without EMPF was 0.5 (20/63) and 0.592 (20/78) (*p* = 0.052), respectively. The eyes in the EMPF group showed a mean decrease of –0.312 logMAR units (*p* = 0.797) in visual acuity following surgery while eyes in the non-EMPF group showed a mean of 0.272 logMAR units' improvement (*p* = 0.008) following surgery. By the end of the final follow-up visit, a significant decrease in visual acuity was noted in eyes with LMH and EMPF who were managed conservatively (*p* = 0.021). Eyes with LMH and no EMPF who were managed conservatively showed a minimal worsening in visual acuity.

Changes in the visual acuity in the two groups before and after surgery is described in Table 4.

##  DISCUSSION 

The use of spectral domain OCT has allowed us to visualize the presence of substantive material on the epiretinal surface in the LMH and the FTMH which we describe as EMPF or LHEP as described by Pang et al.^[3]^ In this article, we studied the clinical and OCT features and surgical outcomes of LMHs with and without EMPF.

The findings in this study suggest that in LMHs, EMPF formation was accompanied by ellipsoid layer loss, a wider than normal macular hole diameter, deep retinal defects, and the presence of IS-OS defects in large-sized MHs. The ERM was not present in all the cases of EMPF. The EMPF was yellowish in color and connected to the retinal tissue within the hole. Taken together, these findings suggested that EMPF could be a secondary event following LMH formation and is usually accompanied with deep outer retina involvement. Also, histological studies have shown absence of the inner retinal tissue within the epiretinal tissue.^[2]^ Many theories were proposed for the development of EMPF in LMH; however, none are conclusive.^[3,4,10]^ The findings of this study reinforce an alternate theory for the EMPF formation. According to this theory, EMPF originates secondary to the defects in the ellipsoid zone which then allows the retinal pigment epithelial cells to migrate along the walls of the MH and then onto the retinal surface and finally leading to EMPF and ERM formation.

**Table 1:** Clinical and optical coherence tomography findings of patients with and without EMPF in eyes with LMH


**Variable**	**LMH without EMPF (** ***n*** ** = 62)**	**LMH with EMPF (** ***n*** ** = 50)**	***P*** **-value **
Mean age (yr)	72.6 ± 8.25	69.9 ± 13.0	0.316 ${}^{\#}$
Sex (M:F)	26:36	33:17	0.667 ${}^{\#}$
Laterality (RE:LE)	35:27	31:19	0.667 ${}^{*}$
Mean presenting logMAR VA (Snellen equivalent)	0.396 (20/50)	0.518 (20/66)	0.05 ${}^{\#}$
Size of LMH (µm)	715 ± 305	986 ± 471	0.001 ${}^{\#}$
Residual retinal thickness (µm)	144 ± 28.1	102 ± 33.7	< 0.001 ${}^{\#}$
Presence of IS-OS defect (*n*, %)	8 (13)	42 (84)	> 0.999 ${}^{*}$
Size of IS-OS defect (µm)	32.8 ± 101	401 ± 389	< 0.001 ${}^{\#}$
Presence of ERM (*n*, %)	52 (84)	42 (84)	0.667 ${}^{*}$
Eyes undergoing surgery (*n*, %)	11 (18)	7 (14)	0.667 ${}^{*}$
LMH, lamellar macular hole; EMPF, epimacular proliferative tissue; VA, visual acuity; ETDRS, Early Treatment Diabetic Retinopathy Study; IS–OS, inner segment–outer segment; ERM, epiretinal membrane ${}^{\#}$ *P*-value calculated using the Mann–Whitney U-test; ${}^{*}$ *P*-value calculated using the Chi-square test			

**Table 2:** Correlation of EMPF with different clinical and OCT variables


	**Age**	**Sex**	**Pre-op log MAR VA**	**Type of LMH**	**Width of LMH**	**Thickness of residual retinal tissue**	**IS–OS defect**	**Size of the IS–OS defect**	**ERM**	**Post op logMAR VA**	**LMH closure**
EMPF	*r* value	–0.145	–0.243	–0.257	0.327	0.233	–0.641	0.742	0.743	–0.082	–0.641	–0.665
	*p*-value ${}^{\#}$	0.107	0.007	0.004	0.000	0.009	0.000	0.000	0.000	0.367	0.001	0.000

EMPF, epimacular proliferative tissue; VA, visual acuity; LMH, lamellar macular hole; IS–OS, inner segment–outer segment; ERM, epiretinal membrane ${}^{\#}$ *P*-value calculated using Spearman' correlation test												

**Table 3:** Surgical outcomes in eyes with and without EMPF


**Variable**	**Surgery in EMPF cases (** ***n*** ** = 7)**	**Surgery without EMPF cases (** ***n*** ** = 11)**	***P*** **-value**
Pre-op mean logMAR VA (Snellen equivalent)	0.5 (20/63)	0.592 (20/78)	0.052 ${}^{\#}$
MH width (µm)	1232 ± 528	795 ± 252	0.027 ${}^{\#}$
Residual retinal thickness (µm)	82.1 ± 16.3	143 ± 30	0.0004 ${}^{\#}$
Presence of IS–OS defect (*n*, %)	6(86)	2(18)	0.013 ${}^{*}$
Size of IS–OS defect (µm)	808 ± 757	54.4 ± 128	0.002 ${}^{\#}$
Presence of ERM (*n*, %)	6(86)	10(91)	> 0.999 ${}^{*}$
Hole closure achieved (*n*, %)	4(57)	11(100)	0.043 ${}^{*}$
Post op mean logMAR VA (Snellen equivalent)	0.518 (20/130)	0.32 (20/42)	0.001 ${}^{\#}$
VA, visual acuity; EMPF, epimacular proliferative tissue; MH, macular hole; IS–OS, inner segment–outer segment; ERM, epiretinal membrane ${}^{\#}$ *P*-value calculated using the Mann–Whitney U-test; ${}^{*}$ *P*-value calculated using the Chi-square test			

**Table 4:** Visual acuity changes before and after surgery in eyes with and without EMPF


	**Mean Pre-op logMAR VA (Snellen equivalent)**	**Mean Post-op logMAR VA (Snellen equivalent)**	***P*** **-value ${}^{\#}$ **
Surgery in EMPF	0.5 (20/63)	0.812 (20/130)	0.797
Surgery in no EMPF	0.592 (20/78)	0.32 (20/42)	0.008
EMPF, epimacular proliferative tissue; VA, visual acuity; ETDRS, Early Treatment Diabetic Retinopathy Study; MH, macular hole ${}^{\#}$ *P*-value calculated using the Wilcoxon signed rank test			

The prevalence of EMPF in LMH has ranged from 20.5% to 44% in previous studies.^[3,7,8,16]^ In our study, EMPF was noted in 44% of eyes with LMH. This is comparable to that observed with other studies. EMPF was seen more commonly with the degenerative variety of LMH (90%) as compared to the mixed or tractional variety. The presence of EMPF was associated with lower presenting visual acuity, larger MH size, thinner residual retinal tissue, and larger IS-OS defects before operation. As a result, the visual and anatomical outcomes following surgery in these eyes were significantly different from those with no EMPF. Eyes with LMH with EMPF showed no visual acuity gain following surgery. Also, successful anatomic closure of the MH was achieved in only four of the seven (57%) eyes following surgery compared to that of the non-EMPF group where the MH closed in all cases (100%). Similar observations were also noted by Choi et al and Ko et al where the visual outcomes in LHEP group was poorer as compared to the eyes with no LHEP.^[7,16]^ However, Lai et al reported no difference in the visual and anatomic outcomes between the LHEP group and non-LHEP group following surgery.^[8]^ In their study, the largest mean diameter on the horizontal scan of the LMH in eyes with LHEP (98.4 µ) was less than the eyes with no LHEP (146.9 µ). While in our study, eyes with LMH with EMPF (1282 µ) had wider large mean diameters on the horizontal scan as compared to eyes with no EMPF (715 µ). Also, the IS-OS defects were much larger in eyes with EMPF (808 µ) than in eyes with no EMPF (54.4 µ). This would explain the poor visual and anatomic outcomes following surgery in our study.

The recommendations for the surgical repair of eyes with LMH remain controversial.^[15,16]^ While there have been reports with good surgical outcomes,^[17,18]^ there have also been reports that have advised caution with performing vitrectomy in these cases.^[1,2]^ In our series, only 18 of the 112 (16%) eyes with LMH underwent surgery. The rest of the eyes were managed conservatively through observation. In the observation group, 43 of the 94 (46%) eyes, which were managed by observation, showed EMPF on the SD-OCT scans, this category further increased to 63% by the end of the final follow-up visit. Thus, suggesting that solely observing such cases may lead to progression of the outer retinal defects and EMPF formation, ultimately leading to decrease in vision. Consequently, we recommend surgery for eyes with LMH when visual acuity is 6/18 or less, there is the presence of epiretinal membrane causing retinal traction, presence of an intact ellipsoid zone, progression in the size of LMH on follow-up visits or progression to full-thickness within the macular hole.

During surgery in cases that possess LMH with EMPF, it is recommended to peel the proliferative tissue while peeling the ERM. The cellular composition of the EMPF may lead to the recurrence of the ERM formation if not removed completely. However, aggressive peeling of the EMPF may lead to the conversion of the LMH to FTMH as seen in three cases in this study. Care should be taken to not forcibly pull the ERM from the edge of the hole. Applying the least amount of traction as possible may theoretically reduce the possibility of retinal tissue damage or the formation of FTMH. In fact, a few studies have reported a high incidence of FTMH formation after the LHEP was peeled in surgeries for LMH.^[2,3]^ Shiraga et al recommended inversion of the pigment containing proliferative tissue into the LMH to facilitate normalization of the foveal contour;^[21]^ however, we did not practice this technique in any of our cases. We did a conventional ILM peeling extending from arcade to arcade in all our cases with the intention of removing the cellular proliferative tissue where possible without much damage to the retina. In some cases, it is sometimes easier to start peeling by first engaging the ILM not occupied by the ERM, and then removing the ILM along with the ERM.

Our study has several clinical implications. Our study suggests that surgery in eyes with LMH with EMPF have both poor anatomic and visual prognosis. Intervention in eyes with LMH without EMPF/LHEP and without ellipsoid zone disruption can have better visual and surgical prognosis.

Our study has the advantage of having an adequate number of eyes both with and without EMPF in LMHs for evaluation. The descriptive features of eyes with EMPF on OCT confirms the outer retinal damage theory of EMPF origin. The study also describes the surgical outcomes of patients operated for LMH with EMPF. The most significant limitation of our study is its retrospective design in accessing pertinent data for evaluation. Our study was further limited as there was only a single observer evaluating the OCT scans, in addition, only a small number of eyes underwent surgery for management of LMH. Extensive clinical and pathological studies may be required to complement our observations and to provide answers to the questions of the cellular origin of EMPF and the reason for its recurrence following surgery.

From this study, we can conclude that EMPF in LMH has a poor visual prognosis when accompanied with ellipsoid zone disruption. Better functional and anatomical outcomes can be achieved following surgery when LMH are not associated with EMPF and/or ellipsoid zone disruption.

##  Financial Support and Sponsorship

Nil.

##  Conflicts of Interest

There are no conflicts of interest.
